# 4,4′-Diazenediyldipyridinium (4-pyridyldiazen­yl)pyridinium octa­cyanidomolyb­date(V) tetra­hydrate

**DOI:** 10.1107/S160053680802494X

**Published:** 2008-08-13

**Authors:** Wen-Yan Liu, Hu Zhou, Ai-Hua Yuan

**Affiliations:** a School of Materials Science and Engineering, Jiangsu University of Science and Technology, Zhenjiang 212003, People’s Republic of China

## Abstract

The structure of the title complex, (C_10_H_10_N_4_)(C_10_H_9_N_4_)[Mo(CN)_8_]·4H_2_O, consists of 4,4′-diazenediyldipyridinium and (4-pyridyldiazen­yl)pyridinium cations disordered over the same site, an [Mo(CN)_8_]^3−^ anion and four uncoordinated water mol­ecules. The cations (crystallographic symmetry, 2) and the [Mo(CN)_8_]^3−^ anion (crystallographic symmetry, 222) are arranged in an alternating fashion, forming a two-dimensional layered structure through hydrogen bonds. Hydrogen bonds, π–π stacking inter­actions (shortest distance = 4.7872 Å) and van der Waals forces between adjacent layers generate a three-dimensional supra­molecular structure.

## Related literature

For information on octacyanidometalate-based compounds complexes, see: Chelebaeva *et al.* (2008 ▶); Ikeda *et al.* (2005 ▶); Kosaka *et al.* (2007 ▶); Matoga *et al.* (2005 ▶); Prins *et al.* (2007 ▶); Przychodzeń *et al.* (2007 ▶); Wang *et al.* (2006 ▶).
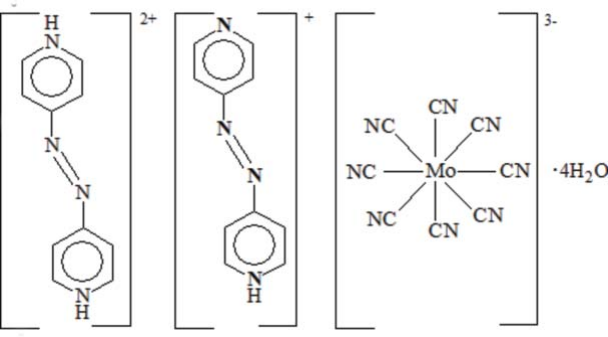


## Experimental

### 

#### Crystal data

(C_10_H_10_N_4_)(C_10_H_9_N_4_)[Mo(CN)_8_]·4H_2_O
*M* *_r_* = 747.60
Orthorhombic, 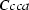

*a* = 16.259 (5) Å
*b* = 12.787 (4) Å
*c* = 15.442 (5) Å
*V* = 3210.5 (18) Å^3^
*Z* = 4
Mo *K*α radiation
μ = 0.47 mm^−1^
*T* = 291 (2) K
0.28 × 0.26 × 0.24 mm

#### Data collection

Bruker APEXII diffractometer
Absorption correction: multi-scan (*SADABS*; Bruker, 2000 ▶) *T*_min_ = 0.879, *T*_max_ = 0.895
13328 measured reflections
1851 independent reflections
1540 reflections with *I* > 2σ(*I*)
*R*_int_ = 0.050

#### Refinement

*R*[*F* ^2^ > 2σ(*F* ^2^)] = 0.032
*wR*(*F* ^2^) = 0.065
*S* = 1.06
1851 reflections
121 parameters
H atoms treated by a mixture of independent and constrained refinement
Δρ_max_ = 0.52 e Å^−3^
Δρ_min_ = −0.28 e Å^−3^

### 

Data collection: *APEX2* (Bruker, 2004 ▶); cell refinement: *SAINT* (Bruker, 2004 ▶); data reduction: *SAINT*; program(s) used to solve structure: *SHELXTL* (Sheldrick, 2008 ▶); program(s) used to refine structure: *SHELXTL*; molecular graphics: *SHELXTL*; software used to prepare material for publication: *SHELXTL*.

## Supplementary Material

Crystal structure: contains datablocks I, global. DOI: 10.1107/S160053680802494X/br2078sup1.cif

Structure factors: contains datablocks I. DOI: 10.1107/S160053680802494X/br2078Isup2.hkl

Additional supplementary materials:  crystallographic information; 3D view; checkCIF report

## Figures and Tables

**Table 1:** Hydrogen-bond geometry (Å, °)

*D*—H⋯*A*	*D*—H	H⋯*A*	*D*⋯*A*	*D*—H⋯*A*
N3—H3*A*⋯O1	0.86 (4)	1.86 (4)	2.675 (3)	157 (3)
O1—H1*A*⋯N2^i^	0.85 (3)	2.06 (3)	2.809 (3)	147 (3)
O1—H1*B*⋯N1^ii^	0.85 (3)	2.40 (3)	3.164 (3)	150 (3)

Symmetry codes: (i) 
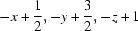
; (ii) 
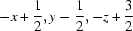
.

**Fig. 1.**
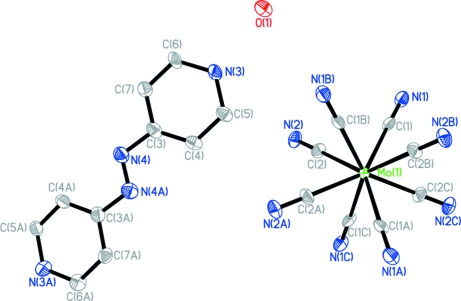
Molecular structure of the title complex showing 30% probability displacement ellipsoids. H atoms are not shown for clarity.

**Fig. 2.**
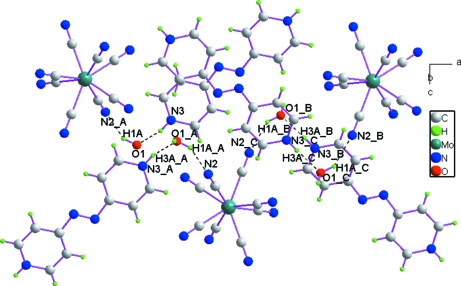
View in the ac plane of the hydrogen-bonding interactions in the title complex. [Symmetry codes: A: 0.5 - x, 1.5 - y, 1 - z; B: 1/2 + x, y, 1 - z; C: 1 - x,1.5 - y, z; D: 0.5 - x, -1/2 + y, 1.5 - z; E: 0.5 - x, 1 - y, z; F: x, 1.5 - y, 1.5 - z; G: 1 - x, 1 - y, 1 - z; H: 1/2 + x, 1.5 - y, -1/2 + z.]

**Fig. 3.**
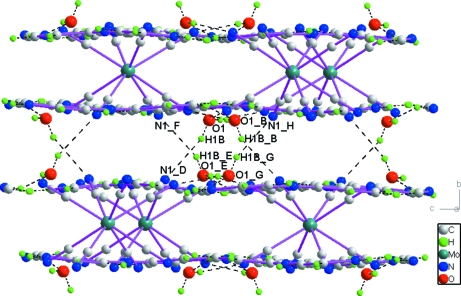
The hydrogen-bonding interactions between adjacent layers in the title complex. [Symmetry codes: A: 0.5 - x, 1.5 - y, 1 - z; B: 1/2 + x, y, 1 - z; C: 1 - x, 1.5 - y, z; D: 0.5 - x,-1/2 + y, 1.5 - z; E: 0.5 - x, 1 - y, z; F: x, 1.5 - y, 1.5 - z; G: 1 - x, 1 - y, 1 - z; H:1/2 + x, 1.5 - y, -1/2 + z.]

## supplementary crystallographic information

### Comment

Recently, the design and synthesis of multifunctional materials with lanthanide octacyanometalate-based metal assemblies are attracting much more interest (Chelebaeva *et al.*, 2008; Przychodzeń *et al.*, 2007; Ikeda *et al.*, 2005; Kosaka *et al.*, 2007; Matoga *et al.*, 2005; Wang *et al.*, 2006). The combination of the octacyanometalate [*M*(CN)_8_]^3-/4-^ (*M* = Mo,W) building blocks with the lanthanide ions plays an important part in the construction of new supramolecular magnetic materials (Prins *et al.*, 2007). In search of a new lanthanide-containing octacyanometalate-based magnet using [Mo^V^(CN)_8_]^3-^and Ce^3+^as the building blocks, we tired to employ 4,4'-azpy (4,4'-azobispyridine) as the primary ligand for coordination. However, the unexpected octacyanomolybdate(V)-based supramolecular complex [H_3_(4,4'-azpy)_2_][Mo(CN)_8_].4H_2_O without Ce^3+^ was obtained instead.

The title complex consists of [H_2_(4,4'-azpy)]^2+^ and [H(4,4'-azpy)]^+^ cations disordered over the same site, [Mo(CN)_8_]^3-^ anion and crystallized water molecules (Fig. 1). It is worth noting that [H_2_(4,4'-azpy)]^2+^ and [H(4,4'-azpy)]^+^ cations are both disordered over the same site.

In the structure, the eight CN groups are all terminal ones and the average distance of Mo—C is 2.1582 Å. The center Mo atom is coordinated by eight cyano groups in a distorted square antiprism. [H_2_(4,4'-azpy)]^2+^ cation, [H(4,4'-azpy)]^+^ cation (crystallographic symmetry, 2), and [Mo(CN)_8_]^3-^ anion (crystallographic symmetry, 222) arranged in an alternating fashion to form a two-dimensional layered structure (Fig. 2) through O1—H1A···N2 and N3—H3A···O1 hydrogen-bonds. Then, a three-dimension supramolecular structure (Fig. 3) was formed through O1—H1B···N1 hydrogen-bonds, π-π packing and Van der Waals forces between adjacent layers.

### Experimental

Single crystals of the title complex were prepared at room temperature in the dark by slow diffusion of anacetonitrile solution (2 ml) containing both Ce(NO_3_)_3_.6H_2_O (21.71 mg, 0.05 mmol) and 4,4'-azpy (9.21 mg, 0.05 mmol) into an acetonitrile solution (20 ml) of [HN(n—C_4_H_9_)_3_]_3_[Mo(CN)_8_].4H_2_O (46.60 mg, 0.05 mmol). After two weeks, pale yellow crystals were obtained.

### Refinement

All non-H atoms were refined anisotropically. The (C,N)H atoms of the 4,4'-azpy molecules were placed in calculated positions with C—H and N—H distances 0.99 Å and 0.92 Å, respectively, with *U*_iso_(H) = 1.2*U*_eq_(C,N). The H atoms of the solvent water molecules were located in a difference Fourier map and refined as riding, with O—H restraints of 0.95 Å, and with *U*_iso_(H) = 1.2*U*_eq_(O).

### Crystal data

**Table:**

(C_10_H_10_N_4_)(C_10_H_9_N_4_)[Mo(CN)_8_]·4H_2_O	*F*_000_ = 1524
*M**_r_* = 747.60	*D*_x_ = 1.547 Mg m^−^^3^
Orthorhombic, *C**c**c**a*	Mo *K*α radiation λ = 0.71073 Å
Hall symbol: -C 2b 2bc	Cell parameters from 13328 reflections
*a* = 16.259 (5) Å	θ = 2.4–27.5º
*b* = 12.787 (4) Å	µ = 0.47 mm^−^^1^
*c* = 15.442 (5) Å	*T* = 291 (2) K
*V* = 3210.5 (18) Å^3^	Block, pale yellow
*Z* = 4	0.28 × 0.26 × 0.24 mm

### Data collection

**Table:**

Bruker SMART APEX CCD diffractometer	1851 independent reflections
Radiation source: fine-focus sealed tube	1540 reflections with *I* > 2σ(*I*)
Monochromator: graphite	*R*_int_ = 0.050
*T* = 291(2) K	θ_max_ = 27.6º
φ and ω scans	θ_min_ = 2.4º
Absorption correction: multi-scan(SADABS; Bruker, 2000)	*h* = −21→21
*T*_min_ = 0.879, *T*_max_ = 0.895	*k* = −16→16
13328 measured reflections	*l* = −19→16

### Refinement

**Table:**

Refinement on *F*^2^	Secondary atom site location: difference Fourier map
Least-squares matrix: full	Hydrogen site location: inferred from neighbouring sites
*R*[*F*^2^ > 2σ(*F*^2^)] = 0.032	H atoms treated by a mixture of independent and constrained refinement
*wR*(*F*^2^) = 0.065	*w* = 1/[σ^2^(*F*_o_^2^) + (0.0235*P*)^2^ + 2.9147*P*] where *P* = (*F*_o_^2^ + 2*F*_c_^2^)/3
*S* = 1.06	(Δ/σ)_max_ < 0.001
1851 reflections	Δρ_max_ = 0.52 e Å^−^^3^
121 parameters	Δρ_min_ = −0.28 e Å^−^^3^
Primary atom site location: structure-invariant direct methods	Extinction correction: none

### Special details

**Table:**

Geometry. All e.s.d.'s (except the e.s.d. in the dihedral angle between two l.s. planes) are estimated using the full covariance matrix. The cell e.s.d.'s are taken into account individually in the estimation of e.s.d.'s in distances, angles and torsion angles; correlations between e.s.d.'s in cell parameters are only used when they are defined by crystal symmetry. An approximate (isotropic) treatment of cell e.s.d.'s is used for estimating e.s.d.'s involving l.s. planes.
Refinement. Refinement of *F*^2^ against ALL reflections. The weighted *R*-factor *wR* and goodness of fit *S* are based on *F*^2^, conventional *R*-factors *R* are based on *F*, with *F* set to zero for negative *F*^2^. The threshold expression of *F*^2^ > σ(*F*^2^) is used only for calculating *R*-factors(gt) *etc*. and is not relevant to the choice of reflections for refinement. *R*-factors based on *F*^2^ are statistically about twice as large as those based on *F*, and *R*- factors based on ALL data will be even larger.

### Fractional atomic coordinates and isotropic or equivalent isotropic displacement parameters (Å^2^)

**Table:**

	*x*	*y*	*z*	*U*_iso_*/*U*_eq_	Occ. (<1)
C1	0.39870 (13)	0.84531 (17)	0.79366 (15)	0.0382 (5)	
C2	0.45460 (14)	0.83539 (19)	0.63923 (15)	0.0427 (5)	
C3	0.41341 (15)	0.62509 (19)	0.31466 (17)	0.0461 (6)	
C4	0.43896 (15)	0.63527 (19)	0.40018 (17)	0.0447 (6)	
H4	0.4948	0.6385	0.4132	0.054*	
C5	0.38109 (14)	0.64066 (18)	0.46624 (17)	0.0450 (6)	
H5	0.3982	0.6474	0.5234	0.054*	
C6	0.27215 (16)	0.62570 (19)	0.36140 (16)	0.0464 (6)	
H6	0.2163	0.6225	0.3484	0.056*	
C7	0.33002 (14)	0.62029 (19)	0.29530 (16)	0.0441 (6)	
H7	0.3129	0.6135	0.2381	0.053*	
Mo1	0.5000	0.7500	0.7500	0.02552 (10)	
N1	0.34570 (12)	0.89415 (16)	0.81824 (14)	0.0468 (5)	
N2	0.43040 (13)	0.87856 (16)	0.57871 (13)	0.0462 (5)	
N3	0.29771 (12)	0.63591 (16)	0.44687 (13)	0.0437 (5)	
H3A	0.262 (2)	0.639 (3)	0.488 (2)	0.052*	0.75
N4	0.46326 (12)	0.62255 (16)	0.24043 (15)	0.0495 (5)	
O1	0.17847 (11)	0.59099 (14)	0.56028 (11)	0.0457 (4)	
H1A	0.1323 (19)	0.593 (2)	0.535 (2)	0.069*	
H1B	0.1865 (18)	0.530 (2)	0.5803 (19)	0.069*	

### Atomic displacement parameters (Å^2^)

**Table:**

	*U*^11^	*U*^22^	*U*^33^	*U*^12^	*U*^13^	*U*^23^
C1	0.0371 (11)	0.0422 (12)	0.0353 (12)	0.0130 (9)	−0.0141 (9)	−0.0110 (9)
C2	0.0442 (13)	0.0452 (13)	0.0387 (13)	0.0103 (10)	−0.0093 (10)	0.0096 (10)
C3	0.0477 (13)	0.0468 (14)	0.0438 (14)	0.0125 (10)	0.0088 (11)	0.0055 (11)
C4	0.0376 (11)	0.0457 (13)	0.0510 (15)	0.0007 (9)	0.0096 (11)	0.0065 (11)
C5	0.0437 (13)	0.0476 (13)	0.0436 (14)	0.0068 (10)	0.0092 (11)	0.0120 (11)
C6	0.0499 (13)	0.0449 (13)	0.0443 (14)	0.0183 (10)	0.0102 (11)	−0.0136 (10)
C7	0.0453 (13)	0.0482 (14)	0.0388 (14)	0.0139 (10)	−0.0010 (11)	0.0019 (11)
Mo1	0.02788 (16)	0.02590 (16)	0.02278 (16)	0.000	0.000	0.000
N1	0.0505 (12)	0.0483 (11)	0.0417 (12)	0.0182 (9)	−0.0095 (9)	−0.0131 (9)
N2	0.0595 (13)	0.0422 (11)	0.0371 (11)	0.0099 (9)	−0.0119 (10)	0.0020 (9)
N3	0.0438 (11)	0.0452 (11)	0.0420 (12)	0.0206 (9)	0.0038 (9)	−0.0123 (9)
N4	0.0492 (10)	0.0498 (11)	0.0496 (13)	0.0047 (9)	0.0063 (11)	−0.0067 (10)
O1	0.0429 (9)	0.0484 (10)	0.0459 (11)	0.0129 (8)	0.0064 (8)	0.0155 (8)

### Geometric parameters (Å, °)

**Table:**

C1—N1	1.130 (3)	C6—H6	0.9300
C1—Mo1	2.157 (2)	C7—H7	0.9300
C2—N2	1.155 (3)	Mo1—C1^i^	2.157 (2)
C2—Mo1	2.159 (2)	Mo1—C1^ii^	2.157 (2)
C3—C7	1.390 (3)	Mo1—C1^iii^	2.157 (2)
C3—C4	1.391 (4)	Mo1—C2^iii^	2.159 (2)
C3—N4	1.404 (3)	Mo1—C2^ii^	2.159 (2)
C4—C5	1.389 (3)	Mo1—C2^i^	2.159 (2)
C4—H4	0.9300	N3—H3A	0.86 (4)
C5—N3	1.390 (3)	N4—N4^iv^	1.231 (4)
C5—H5	0.9300	O1—H1A	0.85 (3)
C6—N3	1.390 (3)	O1—H1B	0.85 (3)
C6—C7	1.390 (3)		
			
N1—C1—Mo1	178.5 (2)	C1^ii^—Mo1—C2^iii^	77.12 (10)
N2—C2—Mo1	178.1 (2)	C1^iii^—Mo1—C2^iii^	72.59 (9)
C7—C3—C4	120.0 (2)	C1^i^—Mo1—C2	77.12 (10)
C7—C3—N4	112.7 (2)	C1—Mo1—C2	72.59 (9)
C4—C3—N4	127.2 (2)	C1^ii^—Mo1—C2	74.20 (9)
C5—C4—C3	120.0 (2)	C1^iii^—Mo1—C2	142.59 (9)
C5—C4—H4	120.0	C2^iii^—Mo1—C2	75.23 (13)
C3—C4—H4	120.0	C1^i^—Mo1—C2^ii^	142.59 (9)
C4—C5—N3	120.0 (2)	C1—Mo1—C2^ii^	74.20 (9)
C4—C5—H5	120.0	C1^ii^—Mo1—C2^ii^	72.59 (9)
N3—C5—H5	120.0	C1^iii^—Mo1—C2^ii^	77.12 (10)
N3—C6—C7	120.0 (2)	C2^iii^—Mo1—C2^ii^	140.02 (13)
N3—C6—H6	120.0	C2—Mo1—C2^ii^	119.25 (14)
C7—C6—H6	120.0	C1^i^—Mo1—C2^i^	72.59 (9)
C3—C7—C6	120.0 (2)	C1—Mo1—C2^i^	77.12 (10)
C3—C7—H7	120.0	C1^ii^—Mo1—C2^i^	142.59 (9)
C6—C7—H7	120.0	C1^iii^—Mo1—C2^i^	74.20 (9)
C1^i^—Mo1—C1	80.44 (12)	C2^iii^—Mo1—C2^i^	119.25 (14)
C1^i^—Mo1—C1^ii^	143.57 (12)	C2—Mo1—C2^i^	140.02 (13)
C1—Mo1—C1^ii^	111.19 (13)	C2^ii^—Mo1—C2^i^	75.23 (13)
C1^i^—Mo1—C1^iii^	111.19 (13)	C5—N3—C6	120.0 (2)
C1—Mo1—C1^iii^	143.57 (12)	C5—N3—H3A	120 (2)
C1^ii^—Mo1—C1^iii^	80.44 (12)	C6—N3—H3A	120 (2)
C1^i^—Mo1—C2^iii^	74.20 (9)	N4^iv^—N4—C3	111.4 (3)
C1—Mo1—C2^iii^	142.59 (9)	H1A—O1—H1B	109 (3)

Symmetry codes: (i) *x*, −*y*+3/2, −*z*+3/2; (ii) −*x*+1, *y*, −*z*+3/2; (iii) −*x*+1, −*y*+3/2, *z*; (iv) −*x*+1, *y*, −*z*+1/2.

### Hydrogen-bond geometry (Å, °)

**Table:**

*D*—H···*A*	*D*—H	H···*A*	*D*···*A*	*D*—H···*A*
N3—H3A···O1	0.86 (4)	1.86 (4)	2.675 (3)	157 (3)
O1—H1A···N2^v^	0.85 (3)	2.06 (3)	2.809 (3)	147 (3)
O1—H1B···N1^vi^	0.85 (3)	2.40 (3)	3.164 (3)	150 (3)

Symmetry codes: (v) −*x*+1/2, −*y*+3/2, −*z*+1; (vi) −*x*+1/2, *y*−1/2, −*z*+3/2.
